# An Open-Source 7-DOF Wireless Human Arm Motion-Tracking System for Use in Robotics Research

**DOI:** 10.3390/s20113082

**Published:** 2020-05-29

**Authors:** Almas Shintemirov, Tasbolat Taunyazov, Bukeikhan Omarali, Aigerim Nurbayeva, Anton Kim, Askhat Bukeyev, Matteo Rubagotti

**Affiliations:** 1 School of Engineering and Digital Sciences, Nazarbayev University, Nur-Sultan Z05H0P9, Kazakhstan; aigerim.nurbayeva@nu.edu.kz (A.N.); a.kim@nu.edu.kz (A.K.); askhat.bukeyev@nu.edu.kz (A.B.); matteo.rubagotti@nu.edu.kz (M.R.); 2 Department of Computer Science, National University of Singapore, Singapore 117417, Singapore; ttaunyazov@nu.edu.kz; 3 School of Electronic Engineering and Computer Science, Queen Mary University of London, London E1 4NS, UK; bukeikhan.omarali@gmail.com

**Keywords:** human arm motion-tracking, inertial sensing and data processing, robot teleoperation, open-source design

## Abstract

To extend the choice of inertial motion-tracking systems freely available to researchers and educators, this paper presents an alternative open-source design of a wearable 7-DOF wireless human arm motion-tracking system. Unlike traditional inertial motion-capture systems, the presented system employs a hybrid combination of two inertial measurement units and one potentiometer for tracking a single arm. The sequence of three design phases described in the paper demonstrates how the general concept of a portable human arm motion-tracking system was transformed into an actual prototype, by employing a modular approach with independent wireless data transmission to a control PC for signal processing and visualization. Experimental results, together with an application case study on real-time robot-manipulator teleoperation, confirm the applicability of the developed arm motion-tracking system for facilitating robotics research. The presented arm-tracking system also has potential to be employed in mechatronic system design education and related research activities. The system CAD design models and program codes are publicly available online and can be used by robotics researchers and educators as a design platform to build their own arm-tracking solutions for research and educational purposes.

## 1. Introduction

Human motion-tracking systems are widely used in several application areas, such as teleoperation of robot manipulators, human–robot interaction, medical diagnostics, video entertainment, virtual reality and animation, navigation, and sport [1,2,3]. Motion-tracking systems record the motion of the whole human body or one of its parts (e.g., an arm) using cameras, wearable sensors, or a combination of those, depending on the task and on the environment. Four types of human body motion-tracking systems are currently available [4]: vision-based systems with markers such as CODA (www.codamotion.com), markerless vision-based systems (see, e.g., [5]), robot-guided tracking systems (see, e.g., [6]), and non-vision-based inertial systems (see, e.g., [3,7,8]). The systems in the first three categories are not completely wearable or portable, and they normally require the presence of grounded devices, ambient light conditions, and/or structured environments [9,10].

Inertial motion-tracking systems are mostly designed as wearable onto human limbs and body, and consist of a combination of small-scale inertial measurement units (IMUs), with each IMU typically containing a tri-axis accelerometer, a tri-axis gyroscope, and a tri-axis magnetometer sensors, for providing the 3D pose of the corresponding human body segment [7,11,12,13]. IMUs transmit processed or raw sensor measurement data to a control PC for further data-fusion and human-motion visualization. Compared to other human motion-tracking systems, inertial systems present the advantages of not needing any light source, of being highly portable, and of being suitable for a wide range of environments. However, they have the drawback of not providing absolute position 35 measurements, and thus suffer from drifts.

The initial development of inertial systems has focused on the 3D position and orientation tracking of a human upper limb with respect to a fixed shoulder joint, representing upper arm and forearm within a two-segment arm model [7,14,15,16]. Numerous algorithms based on Kalman filter or complementary filters were proposed for processing IMU measurement data and for IMU sensor drift reduction [3,16,17,18,19,20]. Detailed surveys of the upper limb motion-tracking methods using inertial sensors are presented in [11,12]. From the kinematic modeling point of view, a human arm can be modeled as a five degrees-of-freedom (DOF) serial manipulator with geometric constraints, where three DOFs are located in the shoulder joint, one DOF is in the elbow joint and the last DOF represents the pronation/supination motion of the forearm [3,21]. Considering that a tri-axis IMU can track up to three DOFs, the arm motion-tracking with respect to a fixed shoulder joint is usually realized using two 3-DOF IMUs placed on the upper arm and the forearm [3,14,15,16]. However, aiming to reduce the impact of inertial sensor data drifting and exploiting the fact that the fifth DOF contributes only to the hand wrist orientation, the authors of this paper proposed in [22] a novel hybrid combination of a 3-DOF IMU, located on the human upper arm, and a simple mechanical tracker equipped with a 1-DOF potentiometer sensor to measure the elbow’s rotation angle: this led to the design a 4-DOF low-cost upper limb position tracking system as a simplified alternative to classical two 3-DOF IMU motion trackers.

**Figure 1:**
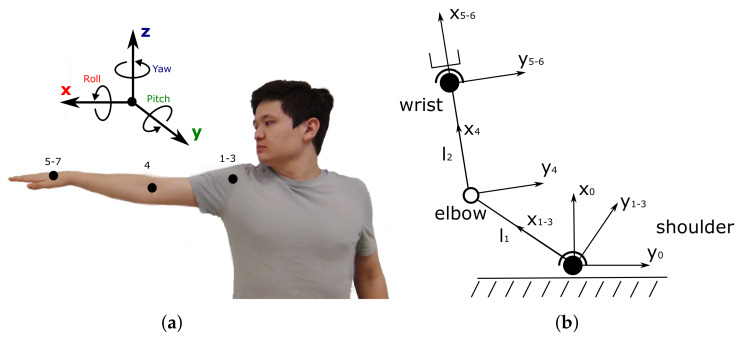
(**a**) A human arm relative to a fixed global frame with indicated DOFs; (**b**) a simplified human arm kinematic model.

For applications requiring full human arm-tracking, including hand pose estimation, a three- segment arm model is typically considered with a third IMU placed on the hand, to form a classical three 3-DOF IMU-based full arm motion-tracking configuration [23,24]. Taking into account that a human arm (with a hand wrist joint) has, in fact, 7 DOFs [21,25] (the reader is referred to Figure 1a for the corresponding graphical representation), this configuration adds significant redundancy to the inertial system, which in turn increases the complexity of the measurement data processing algorithms. Accurate arm-tracking performance is achieved through periodic IMU sensor calibration and assumption of the precise knowledge of the IMU sensor positions on human user’s limbs, and of the user’s limb length dimensions. Therefore, to ensure the immediate deployment of the system and its accurate performance in practice, modern commercially available inertial systems use high-cost IMU sensors and complex system initialization procedures and algorithms for online algorithm parameter identification and tuning. Since most commercially available inertial systems are available as full or upper body motion-tracking solutions, consisting of multiple IMU sensors and supported by proprietary data processing and visualization software suits, e.g., [7], the cost of such systems currently reaches tens of thousands of US dollars, which makes them non-affordable for many individual users and research groups with limited funding. The implementation details of these commercial systems are typically not fully revealed in research publications, as they remain undisclosed for commercial purposes: this makes it difficult, or even impossible, to re-implement similar systems for those research groups that cannot afford buying one of the commercial solutions.

To make the use of arm-tracking systems freely available to academic community, in this paper the authors propose an alternative open-source design concept of a wearable 7-DOF wireless inertial system for a full human arm motion-tracking. Unlike the classical inertial motion-tracking systems with three IMUs, the presented design uses a hybrid combination of two IMUs and one potentiometer, which further develops the author’s initial concept proposed in [22] and aligns with the simplified 7-DOF human arm kinematics. The reduced number of IMUs in the proposed hybrid system design allows the achievement of reasonably accurate tracking performance, sufficient for facilitating the authors’ research on industrial robot teleoperation, by using only an available online popular algorithm for individual IMU raw data processing and a simple quaternion-based arm kinematic model as presented further in the paper.

The majority of the reported publications focus on the development of algorithms for IMU sensor data-fusion. In contrast, this work presents a thorough discussion of the mechanical and electronic design aspects of the proposed hybrid arm motion-tracking system, and details how these aspects have been practically applied to realize a novel ready-to-use system prototype. In this respect, the proposed system provides an additional option to researchers for facilitating robotics research studies and may be also potentially useful in mechatronic system design education and related research activities. For these purposes, the system prototype can be easily replicated using CAD design files and program codes, released online for download at the author’s research lab website https://www.alaris.kz and https://github.com/alarisnu/7dof_arm_tracker and the detailed in the paper off-the-shelf low-cost electronic components.

The paper is organized as follows. Section 2 present a quaternion-based human arm kinematic model, followed by the detailed discussion of the design phases of the proposed arm motion-tracking system in Section 3. Subsequently, Section 4 presents experimental results on accuracy verification of the system prototype, and reports a case study of a UR5 industrial robot-manipulator teleoperation. Finally, Section 5 concludes the paper and outlines potential future work directions.

## 2. Kinematic Modeling of a Human Arm

As mentioned above a human arm can be modeled as a 7-DOF serial kinematic chain [21,25] with 3-DOF shoulder, 1-DOF elbow and 3-DOF spherical wrist joints as schematically shown in Figure 1. Figure 1a shows a human upper limb with dotted points representing the DOFs of the shoulder (spherical 3-DOF joint), of the elbow (1-DOF elbow joint) and of the wrist (3-DOF wrist’s spherical joint), as shown in the corresponding kinematic model in Figure 1b (cf. [3]). Each DOF can be explained in terms of roll, pitch and yaw rotations. If the rotation around an axis does not change the center of gravity of the arm, this corresponds to a roll (i.e., rotation around the *x* axis). The arm flexion and extension movements correspond to the yaw (i.e., rotation around the *z* axis), while the pitch rotation corresponds to the arm abduction and adduction (i.e., rotation around the *y* axis) [26]. The link lengths $l_{1}$ and $l_{2}$ in Figure 1a are associated with upper arm and forearm, respectively.

The human arm kinematic model is formulated using the quaternion representation of spatial rotations [27]. Let quaternion $q_{s}$ denote the shoulder joint orientation and vector $p_{i}=\left(l_{1},0,0\right)$ denote the initial upper arm position when the human arm is fully stretched in a T-pose, as shown in Figure 1a for the right arm case. The elbow joint position $p_{e}$ can then be found as follows:

(1) $$p_{e}=q_{s}⊗p_{i}⊗q_{s}^{*},$$

where ⊗ is the quaternion multiplication operator. The elbow rotation quaternion $q_{e}$ is defined using the elbow angle ϕ as follows:

(2) $$q_{e}=\cos\left(\frac{\phi }{2}\right)+\sin\left(\frac{\phi }{2}\right)i+\sin\left(\frac{\phi }{2}\right)j+\sin\left(\frac{\phi }{2}\right)k,$$

where i, j and k are unit vectors representing the three Cartesian axes. The forearm orientation quaternion is then derived as

(3) $$q_{f}=q_{e}⊗q_{s}.$$

Thus, the wrist position vector $p_{w}$ is expressed as follows:

(4) $$p_{w}=p_{e}+q_{f}⊗\left(l_{2},0,0\right)⊗q_{f}^{*}.$$

The wrist orientation relative to the fixed global frame denoted as quaternion $q_{w}$ is obtained from the wrist orientation measured in its local frame in the form of quaternion $q_{\mathrm{wr}}$ as follows:

(5) $$\begin{array}{c} q_{w}=q_{\mathrm{wr}}⊗q_{f}. \end{array}$$

The proposed forward orientation kinematics of the serial link system is applicable to the right human arm. For the left arm representation, the *x* axis must be rotated 180 degrees in the yaw direction.

## 3. Design of a 7-DOF Arm Motion-Tracking System

The design of wearable body tracking systems requires taking into account many aspects, including ensuring kinematic compatibility, ergonomic human-machine interface, lightweight structure, small and compact design, and cost minimization [28]. In this context, the 3D printing technology was widely adopted in this work as an effective means for system design prototyping with minimum cost and efforts [29], while the system electronics was realized using off-the-shelf low-cost components. The design process of the proposed 7-DOF wireless arm motion-tracking system was carried out in three phases outlined in the following.

### 3.1. Phase 1

**Figure 2:**
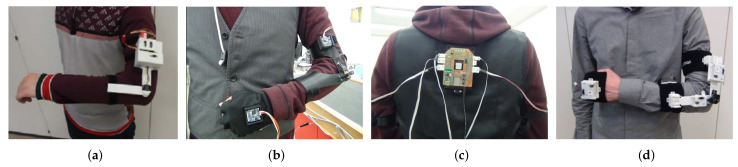
Evolution of the arm motion-tracking system designs: (**a**) the 4-DOF system (Phase 1) [22]; (**b**,**c**) the 7-DOF double-arm system used in [30] (Phase 2); (**d**) the final 7-DOF system (Phase 3) used in [31].

Phase 1 focused on the proof-of-concept verification of the idea of combining an IMU and a potentiometer for arm-tracking. A preliminary 4-DOF arm motion-tracking prototype was designed, as shown in Figure 2a, for upper arm and forearm tracking. Its mechanical design consisted of a 3D printed compact IMU case and a two-link potentiometer holder, with the potentiometer attached on top of the revolute joint of the structure. The IMU case, attached to one link of the holder, would be placed on the upper arm, whereas the other link of the holder would be fixed on the forearm using Velcro strips, so that the holder’s revolute joint axis would align with the user’s elbow joint axis. This would ensure the kinematic compatibility of the system with the user’s arm and allow the potentiometer to measure the elbow joint rotation angle during system deployment. The IMU case and the potentiometer holder had curved bottom designs to allow for a tighter attachment to the operator’s arm, as compared to a non-curved design.

**Figure 3:**
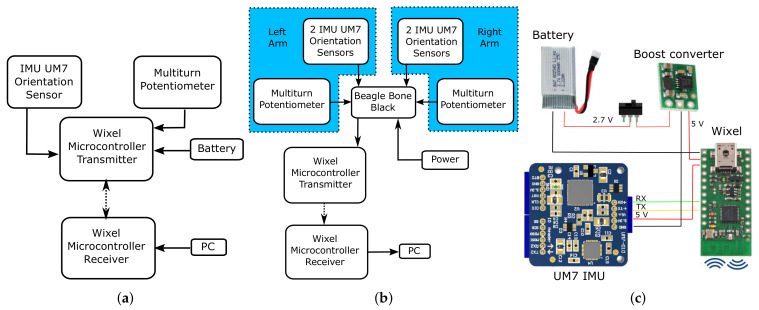
Communication diagrams of the arm motion-tracking system prototypes: (**a**) the 4-DOF system (Phase 1) [22] and (**b**) the 7-DOF double-arm system used in [30] (Phase 2). (**c**) Electronic design of an IMU sensor module (Phase 3).

The embedded system of the tracker was designed by means of a low-cost Redshift Labs UM7-LT orientation sensor (https://redshiftlabs.com.au/product/um7-lt-orientation-sensor/) for raw gyro, accelerometer and magnetometer sensor output. The measurement data were wirelessly transmitted using a pair of Wixel communication modules (www.pololu.com/product/1337) to a control PC for processing through an unscented Kalman filter, as detailed in [22]. This arrangement allowed easy replacement of IMU sensors, to strike the balance between measurement precision and cost, depending on the specific application. The potentiometer that measured the elbow joint angle was also interfaced with the Wixel microcontroller for wireless data transfer to a control PC. Figure 3a demonstrates the hardware architecture of the developed system prototype.

### 3.2. Phase 2

**Figure 4:**
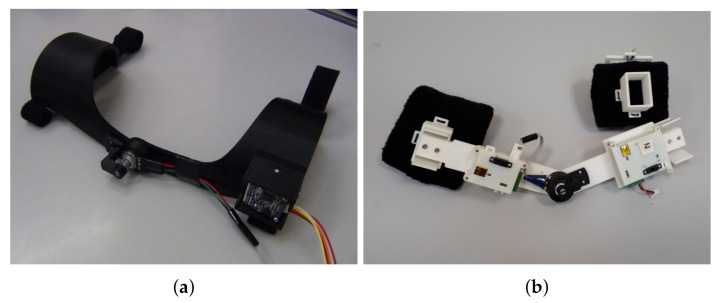
Evolution of the potentiometer and IMU sensor holder designs: (**a**) the 7-DOF double-arm system (Phase 2) and (**b**) the final 7-DOF system (Phase 3).

Phase 2 concentrated on extending the Phase 1 system design to wrist motion-tracking, by using an additional IMU attached to a hand glove, and on improving the ergonomic design of the mechanical structures, as demonstrated in Figure 2b. Specifically, the potentiometer holder was redesigned to ensure more comfort and better aesthetic appearance, as shown in Figure 4a.

At this stage, we decided to experiment with simultaneous double-arm motion-tracking. A powerful microcomputer board based on a BeagleBone Black (BBB) ARM processor, and with an embedded Debian Linux operating system (https://beagleboard.org/black), was chosen as core embedded computing platform. An extension board for the BBB was specifically designed and manufactured for easy interfacing with the system sensors and the Wixel communication module. The board was fixed on the back side of a vest to be worn by the user, as shown in Figure 2c. The board was connected with two identical arm sensor holders and gloves, mounted on the right and left human arms via wired connections. Figure 3b presents the system communication scheme. The Wixel module was set to 20 Hz data transmission rate to ensure stable wireless data transfer to a control PC from all sensors used for double-arm motion-tracking. The system prototype uses the UM7-LT IMU’s built-in embedded extended Kalman filter (EKF) to estimate orientation quaternions, which are then transmitted to a control PC. The data processing algorithm on the control PC side directly combined the received IMU quaternions with the potentiometer measurements, by using the 7-DOF arm kinematic model formulated in Section 2. This simplified the system software and freed the computational resources on the control PC for additional multithreaded execution of real-time robot control algorithms, such as those described in Section 4.5.

### 3.3. Phase 3

The goal of Phase 3 was to finalize the prototype design. The experimental testing of the Phase-2 prototype clearly demonstrated essential design flaws, such as bulky mechanical design, large power supply requirements of the central BBB microcomputer, and its wired connections with the arm-located sensors that was inconvenient for the human operator, and constrained his/her arm motions. Therefore, we decided to employ an independent modular design structure that eliminated the need for a central BBB microcomputer and sensor wiring, and reduced the system power requirements. Moreover, using this approach, the length of the design process was ultimately minimized via several iterations.

Figure 2d demonstrates the Phase-3 arm-tracking system on a human user. Compared with the Phase-2 version of the potentiometer and IMU sensor holder design shown in Figure 4a, the new holder unit shown in Figure 4b is built as a separate device, mounted with clip locks on top of two soft armbands. This greatly simplifies the assembly procedure, as the armbands and the holder can be placed one by one in sequence by the user without external help, which was instead required with Phase-1 and Phase-2 designs. The use of stretched armbands allows their tight placement on the user’s arm, thus preventing circular displacements of the system sensors as a result of skin movement. A potential slight translational motion of the IMU module would not affect the tracker estimates, as the pose estimation algorithms use predefined constant upper arm and forearm lengths $l_{1}$ and $l_{2}$, as outlined in Section 2.

The sensor modules are designed as independent detachable units with integrated small factor Li-Pol power batteries and manual on/off powering via simple mechanical switches for autonomous operation of the units. An electronic design of an individual IMU sensor module, consisting of the UM7-LT IMU interfaced with the Wixel wireless communication module, is shown in Figure 3c. To match the IMU’s and Wixel’s 5V supply voltage level the battery is connected through a Pololu boost voltage regulator (www.pololu.com/product/2115). A potentiometer sensor module has a similar electronic design, except the Wixel board reads an analog signal from a 1 kOhm variable resistance multiturn potentiometer connected through a voltage divider.

**Figure 5:**
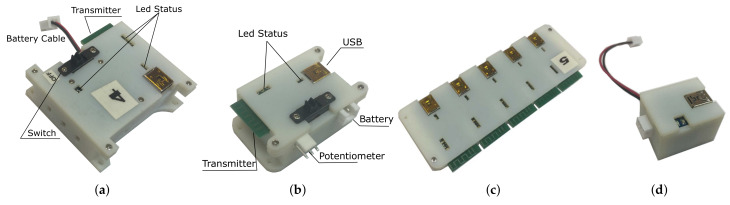
Assembled modules of the final arm motion-tracking system: (**a**) IMU sensor; (**b**) potentiometer; (**c**) data receiver; (**d**) battery charger.

Figure 5a,b demonstrate the assembled sensor modules in 3D printed enclosure boxes. The USB slots of the modules can be easily connected to a PC via a mini-USB cable for updating the Wixel microcontroller software. Several LEDs on each module indicate the internal state of the module, e.g., the activated red LED near the switch indicates that the module is not transmitting data. The sensor modules wirelessly transmit data to five receiving Wixel microcontroller boards, integrated into a common data receiver module shown in Figure 5c. All these five boards are directly connected to a control PC through mini-USB/USB cables. Four of them receive data from the right and left arm IMU sensor modules, i.e., two IMU modules for each arm. The fifth board receives measurement data from one or two potentiometer modules in case of double-arm motion-tracking (the sensor data parsing is implemented at software level). This setting increased the wireless data transmission rate to 100 Hz, which improved on the maximum 20Hz rate of the Phase-2 design. In addition, a dedicated battery charger module embedded into a 3D printed enclosure box was designed as shown in Figure 5d. The charger is powered via a mini-USB - USB cable from a PC, and is connected to the sensor modules through 3-pin and 2-pin connectors. Table 1 summarizes technical specifications of the sensor modules.

The modular final tracker design provides easy replacement of broken sensor modules and/or 3D printed parts in the system prototype, ensures more comfortable wearing of the system on the operator’s arms, and more freedom to his/her movements. In addition, the developed system allows implementation of various data-fusion algorithms for processing raw IMU data on a control PC. To improve the tracking accuracy, the final system prototype uses an open-source gradient-descent algorithm presented in [19], as an alternative to the UM7-LT IMU’s built-in EKF employed in the Phase-2 prototype, as discussed in detail in Section 4.

## 4. Experimental Results

### 4.1. System Sensor Calibration

**Figure 6:**
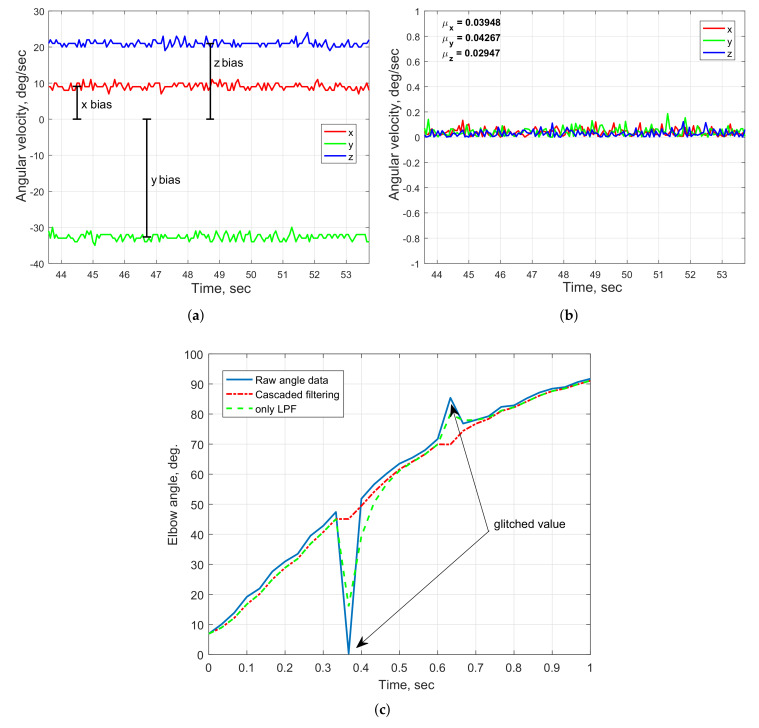
System sensor calibration: UM7-LT IMU gyroscope raw (**a**) and calibrated (**b**) measurements; (**c**) potentiometer data filtering.

The sensor calibration of the arm-tracking system is performed prior to its experimental usage. The IMU requires calibration of its gyroscope and magnetometer, whereas the IMU accelerometer is already available as factory-calibrated. The gyroscope bias can be measured and removed when the IMU is in a stationary position. After sensor calibration, the average amplitude of the gyroscope outputs does not typically exceed 0.2 deg/s in any axis. The comparison of the gyroscope measurements before and after the bias removal is shown in Figure 6a,b. The effect of magnetic distortion is removed by eliminating the bias caused by the presence of electric/electronic devices or ferromagnetic materials in the vicinity of the sensor, and applying the calibration procedure defined in the IMU software interface.

The arm motion-tracking system uses a low-cost 10 kΩ potentiometer that, sporadically, can output unstable analog angle measurements. The glitched values in the raw sensor data can be automatically removed by applying a moving average algorithm (MAA) for selective replacement of the sensor values exceeding a predefined threshold. In addition, a simple low-pass filter (LPF) in form

(6) y[k]=α*x[k]+(1−α)*y[k−1],

with α=0.65 is implemented to eliminate measurement noise which appear due to the wired connection of the sensor with the sensor module. Figure 6c demonstrates the smoothing of the potentiometer elbow angle measurements, in which the cascaded data filtering with the MAA and the LPF ensures more effective outlier point removal compared with the LPF only data processing.

### 4.2. System Tracking Accuracy Evaluation

**Figure 7:**
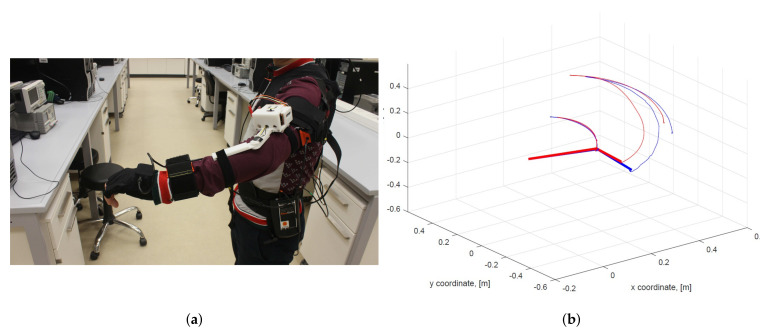
(**a**) The 4-DOF arm tracker (Phase-1) system prototype and Xsens MVN suit on a human user for comparative analysis and (**b**) the captured arm trajectories: blue—the Phase 1 system prototype, red—Xsens MVN suit. Thick lines represent arm segments [22].

The comparative evaluation of the proposed arm motion-tracking concept was performed by conducting arm-tracking tests by a healthy user wearing the Phase-1 system prototype and a state-of-the-art commercial XSens 3D human motion-tracking system (www.xsens.com) serving as a reference, as shown in Figure 7a [22]. The XSens IMU sensors provided the quaternions $q_{s,\mathrm{xsens}}$ and $q_{f,\mathrm{xsens}}$ that were used to estimate the elbow and wrist positions similarly to Equations (1) and (4) [7]. It should be noted that the Xsens upper arm IMU sensor is tilted by −π/2 rad around the $x_{\mathrm{IMU}}$ axis due to the suit layout. The evaluated arm motions included replication of daily activities such as object picking or giving directions. Furthermore, the system was also tested with motions that ensure adequate arm-tracking when executing flexion/extension, abduction/adduction and full elbow flexion movements.

**Figure 8:**
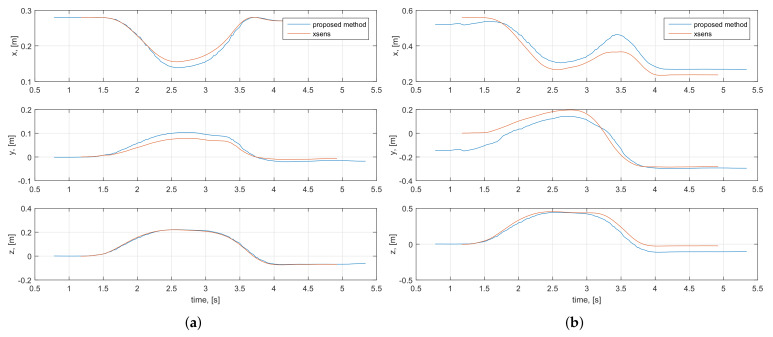
Time evolutions of elbow (**a**) and wrist (**b**) coordinates captured by the Phase-1 system prototype and the Xsens MVN suit [22].

The accuracy evaluation was done using the root mean squared error (RMSE) metric commonly applied for comparative analysis with ground truth references [2,10,32]. During the tests, a total of 60 (x,y,z) elbow and wrist position samples, estimated using both inertial systems, were collected. The comparative analysis resulted in RMSE values of 0.04 m and 0.1 m, respectively, thus indicating higher correlation between the measured elbow positions compared to the wrist trajectories. This is also seen in the example arm motion measurements illustrated in Figure 7b and Figure 8 with the comparison of user’s estimated elbow and wrist coordinates, as well as the overall 3D motion trajectories. In general, the arm trajectories measured by the proposed arm-tracking system closely replicate those of the XSens system, and the comparatively larger wrist position deviations can be interpreted as the result of mismatched initialization procedures. During the T-pose initialization phase, the Xsens system was initialized assuming the full user’s arm extension, i.e., considering the elbow joint angle as ϕ=0, whereas the proposed arm-tracking system was initialized using the actual potentiometer reading of the elbow joint angle, which was in general ϕ≠0. The slight timing mismatch in the graphs was because data were measured at different rates by the two systems in parallel. Furthermore, it was also noticed that the system performance slightly deteriorated during rapid accelerations and near full elbow flexion. This could be attributed to the common fact that the data processing filters of inertial tracking systems are typically tuned for medium accelerations. This problem could be solved by adaptive process noise settings, as proposed in [16].

**Figure 9:**
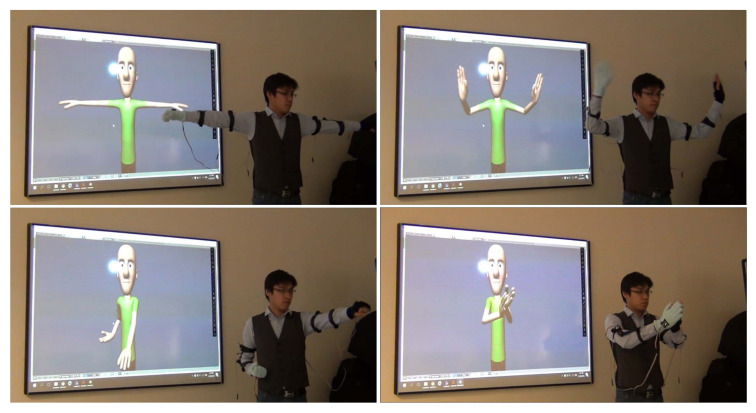
Human operator’s double-arm motion visualization experiment using the Phase 2 arm motion-tracking system at four different time instants.

The system extension to 7-DOF system in Phase 2 was accompanied by captured motion visualizations using an animated humanoid model, implemented in the Blender open-source 3D graphics and animation software (www.blender.org). Figure 9 shows four poses of a system operator wearing the Phase-2 system prototype with no embedded battery support, which are visually very accurately replicated by the animated humanoid model in real time. This confirmed the correctness of the 7-DOF arm kinematic model formulated in Section 2.

### 4.3. Selection of the Data Processing Algorithm

One significant disadvantage of the Phase-2 arm-tracking system with the employed UM7-LT IMU built-in EKF was the fact that it tended to drift due to the IMU magnetometer sensor measurement distortion caused by variations of the magnetic field in close proximity to the computing equipment present in the laboratory. This was noticed using a computer visualization model in which, after several seconds of the system operation, the wrist pose estimations and, hence, the humanoid model, were drifting away from the corresponding operator’s real pose. Initially, drift reduction was attempted by recalibrating the IMU sensors each time before the system usage, as outlined in Section 4.1, and by tuning the process and the measurement noise covariance matrices Q and R of the IMU built-in EKF.

**Figure 10:**
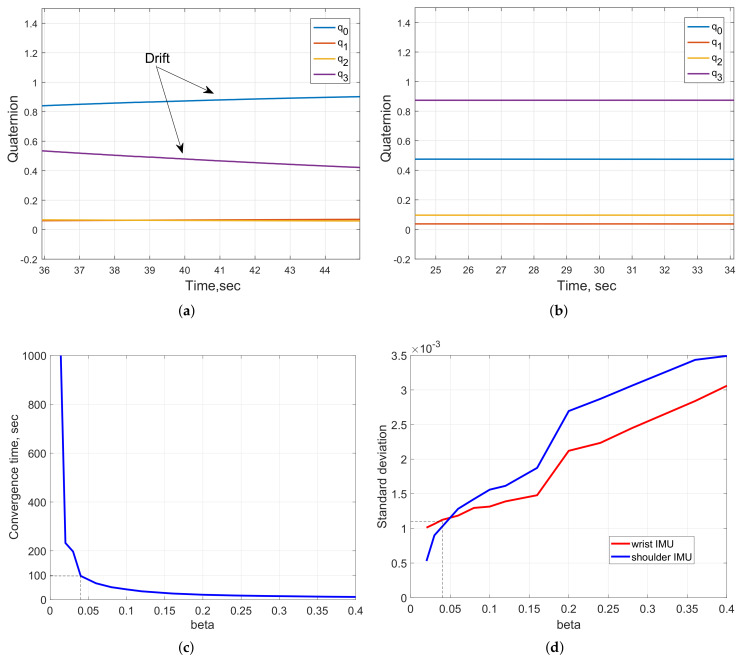
Tuning of data processing algorithms: UM7-LT’s built-in EKF processed quaternion outputs with (**a**) and without (**b**) drift; convergence time (**c**) and standard deviation (**d**) vs. beta gain of GDF [19].

Figure 10a demonstrates the case when the importance given to magnetometer data in the EKF is relatively low, as compared to that of gyroscope data, which resulted in drifting of the IMU quaternion output. In the second case, shown in Figure 10b, the EKF “trusts” in magnetometer readings is increased, which resulted in a more stable sensor output, with a well-calibrated magnetometer. In both the cases, the IMU was in a stationary position. However, this setting with high trust in magnetometer data would not be reliable for prolonged arm-tracking trial experiments, of more than 20 s.

Therefore, as an alternative, it was decided to employ another data processing algorithm for achieving more accurate and stable arm motion-tracking, while reducing the need for frequent IMU tuning. Reviewing various IMU data-fusion algorithms, a popular open-source gradient-descent filter (GDF), developed by Madgwick [19] and available at http://x-io.co.uk/open-source-imu-and-ahrs-algorithms/, was adopted for processing the IMU raw sensor data, due to its more accurate performance, demonstrated in comparative tests using an XSens MTx sensor with a proprietary Kalman-based algorithm. Multiple advantages of GDF, such as low computational cost, integrated compensation of magnetic field variations and easy tuning, led to the adoption of this algorithm by many researchers for further improvement or comparative analysis with their proposed approaches [33,34]. The filter requires only one tuning parameter, i.e., the gain term β, representing the gyro measurement error expressed as the magnitude of a quaternion derivative [19]. While higher values of β (greater than 0.05) result in fast responses of the filter with higher noise amplitude (evaluated using a standard deviation measure for individual IMUs), smaller β gains lead to rapidly increased convergence time, providing more accurate computation, as shown in Figure 10c,d. Based on trial and error tests, a value of β=0.04 was set to ensure accurate and stable tracking performance of the system prototype, compared with the built-in EKF, as demonstrated in the remainder of the paper. However, this led to about 100 s GDF convergence time required for the system prototype initialization, by matching the initial T-pose of the operator (as in Figure 1a). The filter characteristics corresponding to the selected β gain are indicated as dashed lines in Figure 10a,d).

**Figure 11:**
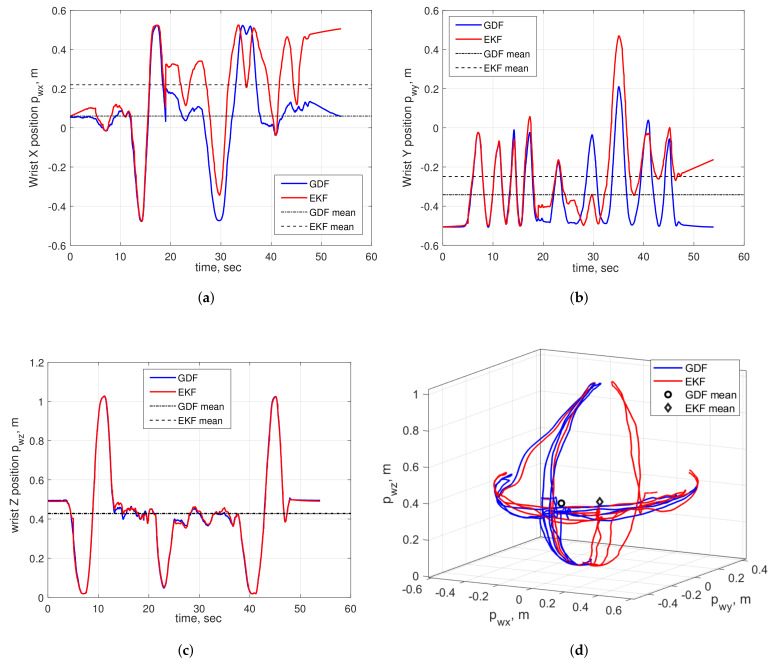
Estimated wrist position trajectories of a human operator performing several left-to-right and up-down arm movements: time evolutions of *x* (**a**), *y* (**b**) and *z* (**c**) wrist coordinates; (**d**) - 3D wrist movement trajectories.

The comparative evaluation of the final system prototype with both the IMU built-in EKF and the GDF data processing algorithms was done using the principle of symmetry of geometrical patterns. Consider a sequence of symmetrical up-down and left-to-right movements repeated by a fully stretched human operator’s arm and centered around the user’s fully stretched-to-front arm pose acting as a starting arm pose. Exploiting the fact that the geometrical center of the overall arm’s wrist movements, computed as a mean value of all trajectory points, in the ideal case lies on the motion’s axis of symmetry and closely correlates with the wrist initial position, the most accurate data processing algorithm can be experimentally identified by analyzing their arm trajectory estimations. At the beginning, an operator wearing the arm motion-tracking assumed the initial T-pose shown in Figure 1a, with the wrist and upper arm IMU modules manually aligned as parallel to each other, for initializing the arm-tracking algorithm after the EKF or GDF convergence. After the tracker initialization procedure was completed, the operator took the starting pose with the user’s wrist positioned at point (0.05,−0.5,0.49) m, defined in the tracker base frame. In this pose, the fully stretched-to-front user’s arm determined the axis of symmetry of the test motions, aligned parallel to axis *y*. Figure 11 illustrates the estimated time evaluations of the *x*, *y* and *z* positions of the operator’s wrist, i.e., $p_{\mathrm{wx}}$, $p_{\mathrm{wy}}$ and $p_{\mathrm{wz}}$, respectively, and the 3D wrist motion trajectory, computed with EKF and GDF. In general, as seen from the above figures, both EKF and GDF are able to closely capture the position and orientation patterns of the operator’s arm test motions and demonstrate close overlapping patterns, especially when estimating $p_{\mathrm{wz}}$, as shown in Figure 11c. This is also confirmed by the approximately equal standard deviations of EKF and GDF estimated trajectories, summarized in Table 2. However, the gradual increase of deviations of the EKF estimated $p_{\mathrm{wx}}$ and $p_{\mathrm{wy}}$ trajectories, demonstrated in Figure 11a,b, indicate the effect of the magnetic field variations affecting the magnetometer measurements, causing, in turn, distortion of heading estimations about the yaw axis [34]. This effect is clearly visible in the 3D wrist trajectory graph in Figure 11d, where one can observe the dynamic heading distortion of the wrist trajectory estimated with the EKF after the first sequence of operator’s left-right up-down arm movements. On the other hand, the GDF estimated trajectory corresponding to the same arm motions remains stable over time and, as a result, accurately captures the operator’s real 3D movements, as can be also observed in Figure 11d. This conclusion is also supported by the statistical analysis of the estimated user’s wrist trajectories summarized in Table 2 (the trajectory mean points are also graphically illustrated in Figure 11). As can be seen in the table, the geometrical center of the GDF estimated trajectory, defined as the mean point (0.062, −0.342, 0.427), is significantly closer to the test motion’s axis of symmetry and the wrist’s starting position (0.05,−0.5,0.49) than the EKF mean point (0.22,−0.249,0.429) with 316%, 18% and −0.4% difference, respectively.

### 4.4. Pick-and-Place Motion Analyses

The proposed arm motion-tracking system was designed with the purpose of facilitating the authors’ research on the real-time teleoperation control of a robot-manipulator, aiming to developing an intuitive robot telemanipulation systems, by closely replicating the human operator’s arm motions. Therefore, repeatability and reliability analyses of the Phase-3 prototype were conducted in the context of a larger study that was approved by the university ethics committee, by adapting a typical object pick-and-place experimental procedure, in which a user repeatedly picks and places an object, e.g., a plastic bottle, from one fixed position to another. Due to the employed cyclic motions and the presence of static phases of the user arm with theoretical zero velocity while picking and placing the object to/from a table, the so-called *zero-velocity update* approach was employed to correct the residual bias errors in IMU angular velocity and linear acceleration measurements at these time instants, an approach already used in inertial human gait detection systems [35,36].

#### 4.4.1. Repeatability Testing

**Figure 12:**
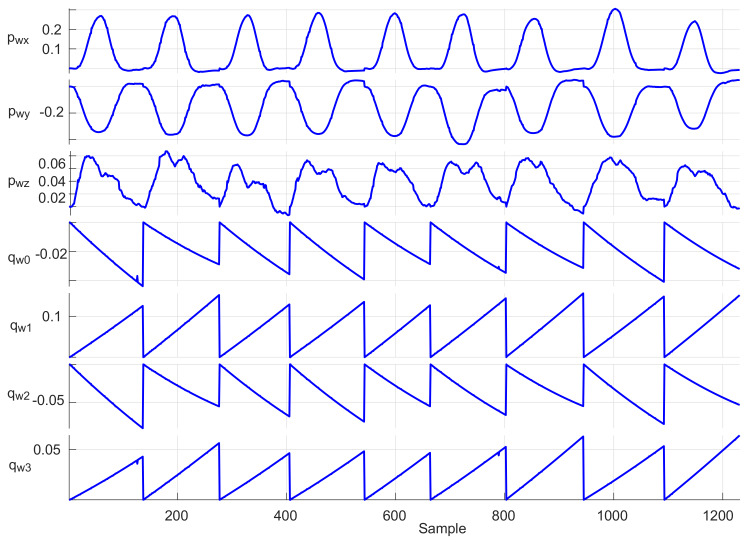
Time evolutions of operator’s wrist position and orientation trajectories for a sequence of nine bottle pick-and-place operations.

Adopting the procedure described in [37], data from nine motions sequences consisting of changing a bottle position on table by a single user were recorded: each sequence contained about 140 data samples of the user wrist positions (*x*, *y*, *z* coordinates) and four orientation quaternion components, i.e., about 1250 data samples in total, as shown in Figure 12. The repeatability test was performed by combining data into a three-dimensional data array $X_{ijk}$, i=1,…N, j=1,…,9, and k=1,…,7 to specify the *i*th position or orientation sample in the *j*th trial for the *k*th parameter. The minimum and maximum of test data were used to determine the range of each parameter as follows:

(7) $$R_{k}=\max_{j}\left({\bar{X}}_{jk}\right)-\min_{j}\left({\bar{X}}_{jk}\right),$$

where

(8) $${\bar{X}}_{jk}=1/N\sum_{i=1}^{N}X_{ijk}.$$

**Figure 13:**
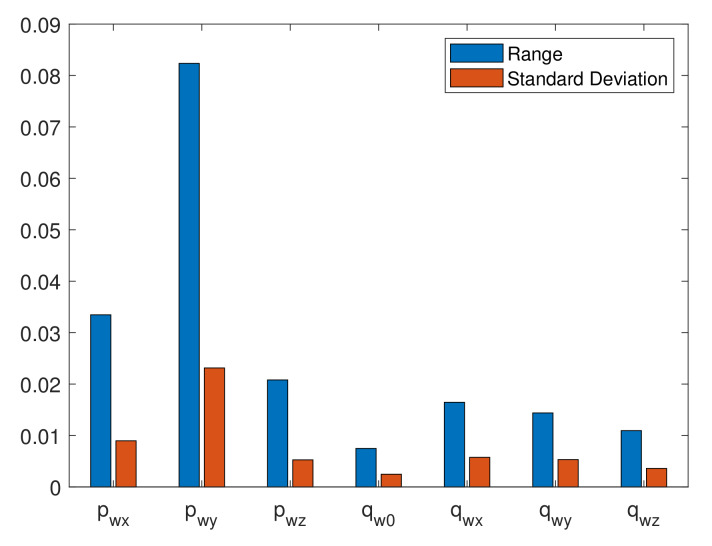
Average range and standard deviation for each parameter.

The individual ranges $R_{k}$ for each sequence, together with the standard deviations (SDs) of the $X_{jk}$ values, are presented in Figure 13. For all test trials, the total average range and average SD, respectively, were obtained as 0.0152 m and 0.0055 m for the user wrist positions, and 0.0042 and 0.0019 for the wrist orientations represented as unit quaternions. These indicators demonstrate the high measurement repeatability of the proposed system while executing the same routine tasks.

#### 4.4.2. Reliability Analysis

The system reliability was assessed using an intraclass coefficient (ICC) for evaluating the variability between measurements. ICC values close to one show a high value of internal consistency [37]. According to [38], ICC values between 0.75 and 0.9 can be considered indicators of a “good” reliability, while values greater than 0.90 indicate “excellent” reliability.

The system reliability analysis was performed by computing the ICC value between two randomly selected trials (out of nine) per parameter, and further repeating the task for all seven parameters. This has been repeated 10,000 times. As a result, ICC values for all trials in the 0.814–0.941 range were obtained as listed in Table 3, with an overall average of 0.871. This indicated high motion-tracking reliability for the proposed system prototype.

### 4.5. System Application in Robotics Research

The developed arm motion-tracking system prototypes were applied for experimental evaluation of real-time teleoperation control frameworks implemented on an UR5 6-DOF industrial robot-manipulator (www.universalrobots.com) operated from a control PC through a custom-made TCP/IP communication driver. The robot end-effector followed the human operator wrist pose (position and orientation) captured using the 7-DOF arm motion-tracking system in the tracker reference frame and translated to the base frame of the UR5 robot with an appropriate scaling, reflecting the user’s arm and robot link dimensions, and an offset in the *z*-axis direction equal to half the length of the robot link 2.

Initial experiments were conducted using the Phase-2 prototype for direct open-loop and various closed loop control algorithms of the robot. As a result, a new UR robot real-time predictive control approach was proposed that ensured smooth motion of the teleoperated manipulator, as presented in detail in [30]. The accompanying video demonstration is available at the author’s research lab website https://www.alaris.kz and https://youtu.be/VOlzN9W4vVs. This research continued using the final system prototype developed in Phase 3. The UR5 robot was equipped with a Robotiq 3-finger adaptive gripper (www.robotiq.com/products/industrial-robot-hand) and controlled using a newly formulated nonlinear model predictive control (NMPC) system that allowed the manipulator end-effector to track the operator’s wrist pose in real time and, at the same time satisfying joint velocity and acceleration limits, avoiding singular configurations and workspace constraints, including obstacles. The detailed formulation of the NMPC control framework and its implementation for the case study of the UR5 robot semi-autonomous teleoperation with obstacle avoidance is reported in [31]. The gripper control was realized using a low-cost force sensitive resistor sensor attached to the operator’s glove and interfaced with the wrist IMU sensor module of the system prototype for sensor signal transmission to a control PC, via the module’s integrated Wixel communication board. The sensor pressing/releasing with the operator fingers activated the gripper closing/opening through a simple thresholding algorithm.

**Figure 14:**
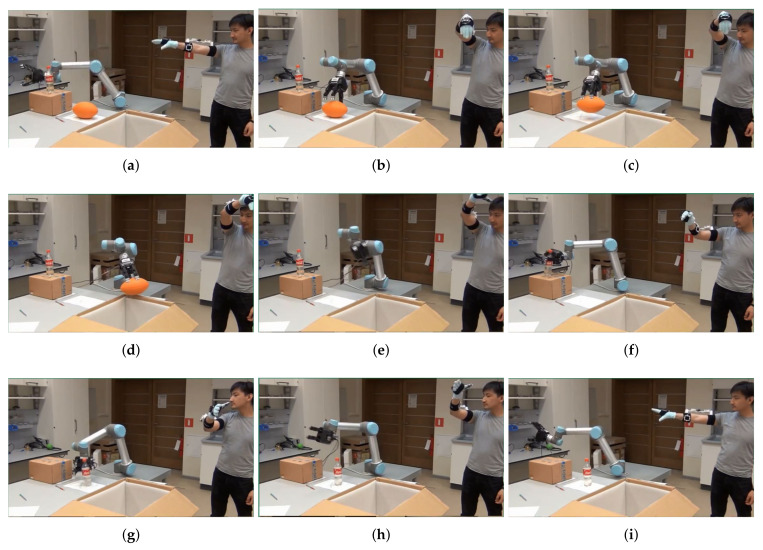
Video frames at nine different time instants during the robot teleoperation experiment using the final arm motion tracking system prototype: the operator assumes the T-pose for initializing the arm motion-tracking system (**a**) and aligning it with the robot, the robot follows the operator’s arm reference (**b**), grasps (**c**), carries towards (**d**) and drops (**e**) the orange ball into an carton box near the table; next the robot moves and grasps the bottle (**f**), places the bottle at the ball’a original position on the table (**g**), and returns to the starting position (**h**,**i**).

Figure 14 presents a sequence of camera shots demonstrating a pick-and-place task execution with the teleoperated robot. An orange ball and a plastic bottle placed on top of a small carton box were chosen a target objects for the experiment. At first, the operator, wearing the arm motion-tracking system and the gripper control glove, assumes the T-pose, as shown in Figure 14a, to initialize the arm-tracking system and align the robot with the arm. Then, the operator moves his/her arm toward the orange ball without hitting the bottle and the box on its way. Figure 14b,c demonstrate the robot accurately following the human hand reference and grasping the ball. Subsequently, the robot carries the ball towards an open carton box near the table in Figure 14d, and drops the ball into the box in Figure 14e. Next, as shown in Figure 14f, the robot moves back towards the bootle and grasps it, places the bottle on the ball’s original position on the table (Figure 14g), and returns to the starting position, as can be seen in Figure 14h,i.

Overall, the experiment confirmed the research hypothesis that the robot can be accurately and intuitively teleoperated in real time by a human operator wearing the developed arm motion-tracking system for quick execution of pick-and-place tasks. The operation time lag of the robot was estimated as, on average, 50 ms when the operator’s movements were compatible with the robot velocity and acceleration limits. In case of fast operator motions, the robot could still follow the reference, but with larger lag. For comparative purposes, the same object pick-and-place tests were conducted using the robot’s native teach pendant. As a result, the experimental task performed by the teleoperated robot was completed four times faster compared with the standard manual robot control method. The video demonstration of this work is available at https://www.alaris.kz and https://youtu.be/N6Urs0l6XAA. Similarly, this work can be easily extended to teleoperating other types of robots, e.g., assistive robotic arms, and/or human–robot interaction research scenarios, using the developed arm motion-tracking system.

## 5. Conclusions and Future Work

This paper has presented in detail the development phases of the hybrid design concept of a 7-DOF wireless human arm motion-tracking system. The reported experimental case study on UR5 robot teleoperation demonstrated the applicability of the developed arm-tracking system robotics research, allowing the authors to bypass the need for high-cost commercial inertial tracking solutions. The system design employs off-the-shelf inexpensive electronic components and 3D printing technology for prototyping and allows the implementation of available open-source or self-developed data-fusion algorithms, ensuring low-cost and straightforward replicability of the system prototype, which can be easily used in mechatronic system design oriented activities. The 3D design models of the system module cases and module holders, created using the SolidWorks CAD software, and program code are made freely available for download from the authors’ research lab website https://www.alaris.kz and https://github.com/alarisnu/7dof_arm_tracker. The simple system design released for public use can be easily reproduced and modified by robotics researchers and educators as a design platform to build their own arm-tracking solutions for research and educational purposes.

More work needs to be conducted in future on improving the estimation accuracy of the proposed human arm-tracking system, which can be achieved through incorporating arm kinematic constraints, e.g., similarly to the results presented in [15,16], and integrating system self-calibration for eliminating the need for manual sensor alignment, e.g., by using the approach presented in [39]. At present, the developed system is operated under the assumption of a still shoulder joint during arm motion measurements. In practice a human operator often moves his/her body during robot teleoperation tasks, thus determining a motion of the shoulder. The system accuracy for this case must be further investigated by using more accurate quaternion-based joint update filtering techniques, incorporating linear acceleration in addition to gravity measurements (see, e.g., [7]). More comprehensive experiments should also be performed to further evaluate the usability of the system for advanced robot teleoperation tasks, in terms of user comfort and ergonomics, and the system applicability to more inclusive scopes of users, e.g., for joint angle monitoring in rehabilitation practice of after-stroke patients, e.g., similarly to work [40].

## Figures and Tables

**Table 1:** Specifications of the Arm Motion-Tracking System Sensor Modules.

Sensor Module	IMU	Potentiometer
Battery capacity	600 mAh	600 mAh
Supply voltage	5 V	5 V
Time duration of one charge	∼2 h	∼2.5 h
Wireless transmission frequency	up to 100 Hz	up to 100 Hz
Programming interface	mini-USB	mini-USB
Dimensions	45×50×15 mm	40×45×15 mm

**Table 2:** Statistical Analysis of the EKF and GDF Estimated Wrist Trajectories.

Parameter	Starting Point, m	Mean (m)		SD (m)		Mean Difference, % (‖GDF-EKF‖/Initial Point)
		GDF	EKF	GDF	EKF	
$p_{\mathrm{wx}}$	0.05	0.062	0.220	0.215	0.239	316
$p_{\mathrm{wy}}$	−0.5	−0.342	−0.249	0.178	0.200	18
$p_{\mathrm{wz}}$	0.49	0.427	0.429	0.228	0.228	0.4

**Table 3:** Computed user wrist’s ICCs.

$p_{\mathrm{wx}}$	$p_{\mathrm{wy}}$	$p_{\mathrm{wz}}$	$q_{\mathrm{wx}0}$	$q_{w1}$	$q_{w2}$	$q_{w3}$
0.942	0.912	0.814	0.854	0.89	0.837	0.852
